# Effect of information on geographical origin, duration of transport and welfare condition on consumer’s acceptance of lamb meat

**DOI:** 10.1038/s41598-020-66267-4

**Published:** 2020-06-16

**Authors:** Mariangela Caroprese, Maria Giovanna Ciliberti, Rosaria Marino, Fabio Napolitano, Ada Braghieri, Agostino Sevi, Marzia Albenzio

**Affiliations:** 1 Department of Agricultural, Food, and Environmental Sciences, University of Foggia, Via Napoli 25-71121 Foggia, Italy; 2 School of Agriculture, Forest, Food and Environmental Sciences, Università degli Studi della Basilicata, Via dell’Ateneo Lucano 10, 85100 Potenza, Italy

**Keywords:** Environmental impact, Psychology and behaviour, Sustainability, Animal physiology

## Abstract

Animal production system and welfare conditions can influence consumers’ acceptance, as meat from animals grazing in natural pasture and labelled with information about high standards of welfare is preferred. In addition, geographical origin of food is recently considered one of the main information influencing the consumers’ acceptance. Local products are collectively associated with high quality attributes by the consumers related to shorter transport and good welfare. Lamb meat is considered local and typical food; however, it is common to find in the same market both local and imported lamb meat. The present investigation aimed at understanding the importance of information about geographical origin, transport duration, and welfare condition of lambs for consumers and their actual liking. Moreover, the quality of lamb meat from local and imported animals as affected by short or long transport was assessed. Data demonstrated that both short and long transport did not affect organoleptic quality of meat; this result was corroborated by an absence of both metabolic and immune stressors in long term transport lambs except for haptoglobin, cortisol and glucose. However, the expected and actual acceptability were affected by the information with higher scores for local lamb when information on the geographical origin, transport duration, and welfare condition was provided to the consumers.

## Introduction

Consumers’ quality perception evolves continuously according to different phenomena and cultural scenario. The geographical origin is considered a relevant credence attribute due to the consumers’ ethnocentrism inducing a preference for food originating from the consumers’ own provenance^1^; thus, the perception of food quality is reinforced when the specific producing areas are limited. Investigations on information about origin of food and consumers’ responses demonstrated that the origin/region of production is one of the most important informational cues for the consumer^2^. In particular, a Spanish case study discovered that the quality labels such as Protected Geographical Indications (PGI) is an important factor for purchasing of lamb meat^3^. It has been found that the stronger and more favourable the association of the food with the country, the greater is the level of food success to consumers^4^. In this context, Jordana^5^ has shown that traditional food has good perspectives for growing in the future if appropriate communication, legal protection of collective brands, quality assurance and innovation will be achieved as challenges. For the quality assurance, information about healthiness is considered as one of the main quality attributes influencing the expectance and the overall acceptance of food that encourage the consumers to try them^6^.

In Guerrero *et al*. study^7^ it has been found that consumers consider four important specific dimensions of traditional food products, including familiarity of the product, processing through traditional recipes, sensory properties and the origin of the product. Lamb meat has a familiarity dimension and its sensory properties are specific and linked to the habit of consumers’ actual area^8^. Moreover, for lamb meat, quality attributes are connected to animal grazing in natural pasture, and consumers are attracted by labelling information about welfare and feeding system^9^. Production of lamb meat is relatively international, as a consequence, meat from different countries can be found in the same market, but consumers are often advised of lamb meat origin^10^.

The transport of animals from farm to abattoir causes an inevitable condition of stress in animals according to Broom’s definitions of stress as the state of an individual as regards its attempts to cope with its environment, and as an effect of the environment on an individual that overcomes the animal’s control system and reduces its fitness^11,12^. Exposition to transport stressors involving temperature fluctuations, handling, and mixing with con-specifics^13^, results in an alteration of animals’ homeostasis which is counterbalanced by an increase of the activity of a number of enzymes and hormones^14^. Road transport related-stress could negatively affect the animal performance, being responsible for increased mortality and decreased meat quality and animal welfare^15^ with potentially relevant economic losses^16^.

Our hypothesis was that the information about local lamb production, subjected to short transport and good welfare can influence the overall consumers’ acceptability respect with imported lamb meat, subjected to long transport and poor welfare, reinforcing the lamb meat local market as more ethical and animal welfare friendly. In order to mimic a more real-life consuming experience and for marketing purposes it is interesting to evaluate the impact of non-sensory attributes (i.e. information) on consumer liking^17^. Therefore, the aim of the present study was twofold: i) to assess the welfare conditions and quality of meat from local lambs subjected to a short transport before slaughter (STR), and lambs imported subjected to a long transport time before slaughter (LTR), ii) to investigate the effect of the information about lamb geographical origin, transport duration, and welfare condition on consumers’ perceived, expected and actual acceptability.

## Results and Discussion

### **Preliminary focus group and food purchase decision**

In order to evaluate the interest of consumers regarding the consumption of lamb meat from different geographical origins and with different welfare conditions, a preliminary focus group was carried out. The discussion within the focus groups recognised lamb meat as a product generally consumed in particular occasions (e.g. Christmas, Easter) and events (e.g. dinner with the enlarged family, dinner with friends). The aspects considered relevant in the purchase decision of the people composing the focus groups were classified according to the topics identified during the discussion (Table 1). Three main clusters were identified. The first one concerned the sensory characteristics of lamb meat; in particular, flavour and taste were considered to be highly intense, thus on one hand capable of differentiate the product, on the other potentially inhibiting lamb meat consumption. The age of the lambs at slaughter was also mentioned as a factor indirectly affecting purchase decision as it was deemed capable of influence the sensory characteristics of the product. Another intrinsic characteristic identified by the focus groups as relevant was the healthiness of the product. The second cluster included the geographical origin and the brand of the product. The former may influence purchase decision for safety reasons as the discussion highlighted a higher trust in the national sanitary control processes and transparency as compared with imported lambs. In addition, the focus groups emphasised that imported lambs may undergo long transport, which induces high stress levels, thus potentially compromising the quality of the product. The brand may add on this by enabling the recognition of local products, obtained from animals subjected to very short distance transport, thus deemed safer and higher quality. The focus groups also identified ethical concerns related to the farming systems used to raise the animals and potentially affecting purchase intentions. Free-ranging and ewe-reared animals were perceived as being in higher welfare conditions, in terms of expression of natural behaviour. In addition, a high welfare state of the animals was also considered to be able to positively affect product quality.

**Table 1:** Breakdown of the aspects identified as relevant by the focus groups when discussing about lamb purchase decision.

Aspect	Item
Sensory and health characteristics	Taste
	Age at slaughter as affecting flavour
	Dietetic characteristics
Origin	Geographical origin
	Brand
Ethical concerns	Rearing system (i.e. confinement or free-ranging)
	Suckling system (i.e. ewe reared or artificially reared)

The scores given by 101 consumers to the items identified by the focus groups are reported in Table 2. The item geographical origin received the highest score albeit not significantly different from taste. Previous studies showed that safety and sensory characteristics were the main choice determinants for various food products^18,19^; whereas, the origin was less important and related to the traditional image of locally transformed animal-based products^18,20^. These results are only apparently in contrast with our findings. Such differences can be explained by taking into account the reasons given by the focus groups for indicating the geographical origin as a food choice determinant: trust in the national control process, and negative effects of long transport on meat quality, both related to the overall safety of lamb. In our study the lack of any references to typical and traditional transformation practices was likely due to the fact that lamb is sold as fresh product with little or no process characteristics possibly linked to the its image.

**Table 2:** Scores given to each item included in the Food Choice Questionnaire and affecting lamb purchase decision (means ± SE).

Item	Score^a^
Geographical origin	6.60 + 0.17a
Taste	6.29 + 0.17ab
Rearing system (i.e. confinement or free-ranging)	6.00 + 0.17bc
Suckling system (i.e. ewe reared or artificially reared)	5.54 + 0.17 cd
Brand	5.24 + 0.17d
Dietetic characteristics	4.52 + 0.17e

^a^Mean scores obtained using a 7-point Likert scale (1 = unimportant to 7=very important). Different letters indicate significant differences at P < 0.05.

As also observed for other animal products, ethical concerns played an important role in defining consumer purchase decision, although less than sensory and safety characteristics. In particular, Napolitano *et al*.^21^ noted that the information concerning animal welfare is able to increase the expectations of the consumers about various animal based-products. In addition, the consumers generally tend to assimilate their liking and increase the actual acceptability towards the expectations. However, both sensory characteristics and ethical concerns contribute to the expression of consumer preferences^22^.

As to brand, previous reports indicate that this item tend to show a low rank in affecting consumers purchase decision^18^. This is confirmed for lamb which is commonly sold as an undifferentiated product. Dietetic characteristics were the least scored item. This result can be attributed to the fact that lamb is not part of the regular diet of most Italian consumers, whereas it is predominantly purchased and consumed in particular events and occasions, as also stated in the discussion of the focus groups. Therefore, consumers are possibly aware that this food may have a minor impact on their health status. Results from the focus group were the preliminary to design the experiment on transported lambs and to plan the subsequent consumer test to investigate the effect of the information about lamb geographical origin, transport duration, and welfare condition on consumers’ acceptability.

### Effects of Transport on Blood Parameters

Transport stress is activated by the complex series of operations related to transport and slaughter, and can be measured with both behavioural and physiological indicators. Plasma cortisol and glucose levels have been considered as reliable biomarkers to measure stress responses of farm animals^23–25^. Lambs subjected to long transport resulted in an increase of both haptoglobin and cortisol levels, with a reduction of glucose level (P = 0.007, 0.015, and <0.0001, respectively, Table 3). Individuals exposed to a relatively short-term or long-term stressor can react changing the levels of hormones that modify the availability of substrates, being more readily for subsequent actions. According to Sapolsky *et al*.^26^, the first wave of endocrine response occurs within seconds, and involves secretion of catecholamines, hypothalamic release of CRH and, perhaps 10 sec later, enhanced secretion of pituitary ACTH, pituitary secretion of PRL and (in primates), GH, and pancreatic secretion of glucagon, together with decreased release of GnRH and of pituitary gonadotropins. A second, wave involves the steroid hormones; therefore, in some minutes, GC secretion is stimulated and gonadal steroid secretion declines. Moreover, suboptimal condition of transport, causes also an increase in the blood plasma, of acute phase proteins among which, haptoglobin, serum amyloid A and C-reactive protein^27^. The role of these acute phase proteins regards the defences to diseases and the modulation of inflammatory responses, such as haptoglobin, which removes damaged haemoglobin and has immunomodulatory role during inflammation^28^. Our data showed both increasing of haptoglobin and cortisol in LTR and agreed with Ekiz *et al*.^25^ who found significantly increased cortisol levels after 75 min transport in lambs in comparison to initial levels. On the contrary, Dalmau *et al*.^15^ stated that no effect on serum cortisol was found in lambs transported for 24 h, even if higher faecal cortisol metabolites were found as compared with lambs transported for 1 h. Thus, the hypothesis of accumulative stress in lambs transported for long period was suggested. The secretion of cortisol activates gluconeogenesis by stimulating the liver to produce more glucose to sustain stressful situations starting from fat and proteins^29^. Adenkola and Ayo^14^ found that plasma glucose level tended to increase during transport as a stress response, primarily due to gluconeogenic effect of cortisol^30^. In goats, Kannan *et al*.^23^ reported that glucose concentration began decreasing at 3 h after 2.5 h transportation. In our study LTR resulted in a lower glucose level than STR, probably associated with the higher consumption of glucose as fuel for restoring homeostasis^31^.

**Table 3:** Effects of pre-slaughter short (STR) or long transport (LTR) on lamb blood parameters (Least Squares means ± SEM).

	STR	LTR	SEM	P-value
Haptoglobin, mg/mL	1.00	2.86	0.45	0.007
Cortisol, ng/mL	12.94	21.70	2.40	0.015
Glucose, mg/dL	87.12	64.47	3.34	<0.000
NEFA, μmol/L	347.40	318.13	42.19	0.620
CK, U/L	605.47	510.87	188.86	0.730
Neutrophils, %	39.26	47.38	4.4	0.240
Lymphocytes, %	54.56	42.65	5.56	0.120
Monocytes, %	4.88	6.48	1.3	0.430
Eosinophils, %	0.37	0.41	0.07	0.730
Basophils, %	0.99	1.18	0.09	0.160
N/L	0.81	1.18	0.2	0.230
Haematocrit, %	24.85	23.2	1.9	0.560
PCV, %	33.53	32.62	0.5	0.220

NEFA: non-esterified fatty acid; CK: creatinine kinase; N/L: Neutrophil to lymphocyte ratio; PCV: packed cell volume.

In our study the level of both NEFA and CK, and the white blood cell percentages were not affected by the duration of lamb transport. CK and lactate dehydrogenase (LDH) activity are considered as indicators of muscle damage, high physical activity or trauma occurring during transport^32^. Moreover, NEFA concentration increased concomitantly during long transport^33^ in response to adrenaline release and is considered a good indicator of body fat utilization in stressful condition during transport^29^. The lack of differences between STR and LTR in CK and NEFA levels suggested that lambs recruited in the trial showed minimal muscle trauma and positively activated the metabolic response to transport stress. This result agreed with those obtained from De la Fuente *et al*.^34^ in which no differences in CK between lambs subjected to different transport times were registered. Moreover, the welfare issues assessed at slaughterhouse (Table 4), taking into account the percentage of active animals, ambulatory animals, injuries and lameness, did not evidence differences between STR and LTR lambs, corroborating the lack of strong negative effect of transport condition on lambs.

**Table 4:** Welfare issues (percentage of active animals, ambulatory animals, injuries, lameness, dead ±SEM) of lambs subjected to short pre-slaughter (STR) or long pre-slaughter transport (LTR).

	STR	LTR	P-value
Active animals, %	80 ± 9.09	86.7 ± 10.6	0.580
Ambulatory animals, %	13.3 ± 9.09	20 ± 10.6	0.580
Injuries, %	0	6.6 ± 6.6	0.330
Lameness, %	0	6.6 ± 6.6	0.330
Dead, %	0	0	0.990

A stressful condition can be detected also by the evaluation of the changing of white blood cell (WBC) numbers, packed cell volume (PCV)/haematocrit^13,35^, and the N/L ratio^30^. In our study no changes in white blood cells (neutrophils, lymphocytes, monocytes, eosinophils, basophils, and N/L ratio), haematocrit, and PCV between STR and LTR were registered. In condition of stress, the increase of cortisol was associated to neutrophilia, lymphopenia^23^ and declined in PCV^36^, thus consequently increasing the probability of health problems and reducing the ability of the lambs to cope with transport stress. The changes in PCV could be related to dehydration; however, animals overheated and dehydrated did not registered different PVC than animals at normal temperature and degree of hydration^37^. In certain circumstances PCV can be a useful measure but is not a general welfare measure. In our experiment, transport duration did not affect the haematocrit and N/L ratio, which were within physiological reference values for lambs of a similar age and weight, as reported in previous papers^13,38^. On the whole, physiological response of lambs to duration of transport, was characterized by cortisol, haptoglobin and glucose levels alteration without affecting CK, NEFA and other haematic parameters; thus, demonstrated a lack of any additional stress to the transport.

### Meat quality

No significant differences due to transport duration on lamb meat chemical composition were found (data not shown). Protein content and fat content ranged from 20.03 to 20.48 ± 0.14 (STR and LTR), and from 3.28 to 3.45 ± 0.11 (LTR and STR), respectively.

Colorimetric parameters, mechanical properties and pH of lamb meat are reported in Table 5. The pH value measured at 1 h was lower (P < 0.05) in *longissimus* muscle of LTR lambs compared to STR lambs; however, at 24 h *post mortem* no significant differences were found between groups. No effect of transport duration on colorimetric parameters and mechanical properties was found.

**Table 5:** Effects of pre-slaughter short (STR) or long transport (LTR) on lamb pH, colour and mechanical properties (Least Squares means ± SEM).

	STR	LTR	SEM	P-value
pH	6.59	6.34	0.05	0.003
pH, 24 h	5.68	5.62	0.09	0.640
Lightness, L	47.95	49.86	0.67	0.147
Redness, a	11.89	10.17	0.38	0.078
Yellowness, b	10.47	10.14	0.35	0.525
Chroma, c	15.72	14.62	0.52	0.146
Hue angle, h	41.90	44.27	1.07	0.128
WBS, kg	5.86	5.54	0.19	0.244
Hardness, kg	4.95	5.03	0.24	0.182
Choesiveness	0.13	0.12	0.01	0.425
Springiness, mm	5.93	5.76	0.21	0.335
Gumminess	0.64	0.61	0.04	0.063
Chewinness	3.81	3.47	0.30	0.004

As reported in a previous study^39^, the ultimate meat pH is an indicator of meat quality, since it may affect important quality characteristics, such as tenderness, colour and water holding. It is known that the ultimate pH value is influenced by many factors including pre-slaughter handling, transport, lairage period, *postmortem* treatment, glycogen storage and muscle physiology. In the present study, the different transport duration did not affect the pH at 24 hours *post mortem*. This result could be due to the long lairage period that allowed the animal to replenish muscle glycogen reserves, although previous results on the time and conditions of lairage necessary to allow a recovery of the transportation stress prior to slaughter are controversial. Devine *et al*.^40^ claimed that recovery should be longer than three days, for these authors a single day rest does not allow the replenishment of glycogen loss due to pre-slaughter stress in lambs. On the contrary, Ekiz *et al*.^25^ found similar meat pH level both in meat from lambs subjected to transport and left at lairage for 18 h and meat from non-transported lambs. In the present study, a 12 hours lairage was adequate to reach optimal pH conditions, as also confirmed by the results on meat tenderness and colour. In addition, our results on meat quality are accordingly related to those on blood indicators showing no marked differences between lambs subjected to long or short transport, thus, suggesting that pre-slaughter stressors did not lead to a decline in meat quality.

### Consumers’acceptability test

Table 6 shows the results of perceived, expected and informed acceptability of meat from local lambs subjected to a short transport time before slaughtering with a low impact on animal welfare and from imported lambs subjected to a long transport time before slaughtering with an important impact on animal welfare. No difference between STR and LTR meat was observed for perceived acceptability; this was probably due to the comparable chemical composition and texture profile of meat from STR and LTR, as previously discussed. On the contrary, meat from STR showed a higher expected acceptability than meat from LTR (P < 0.001). In addition, the expected acceptability for STR meat was significantly higher than the liking expressed in blind conditions (P < 0.01), while the expected acceptability for LTR meat was significantly lower than the perceived acceptability (P < 0.05), thus indicating that a disconfirmation took place in both cases. In particular, consumers perceived meat from lambs STR worse than expected (negative disconfirmation); whereas, they found meat from lambs LTR better than expected (positive disconfirmation). Therefore, information about lamb geographical origin, transport duration, and welfare condition had a marked impact on consumer expectancy; indeed, meat from animals subjected to reduced transport stress with local origin were associated with high expected product quality.

**Table 6:** Rating (±SEM) given by the consumer panel during the three consumer/hedonic tests.

	STR	LTR	SEM
Perceived liking (P)	6.85	6.64	0.16
Expected liking (E)	8.19a	4.29b	0.16
Informed liking (I)	7.82a	6.28b	0.14
P-E	−1.34***	2.38***	0.22
	Negative disconfirmation^a^	Positive disconfirmation^b^	
I-P	1.00***	−0.37	0.22
	Assimilation^c^		
I-E	−0.36*	—	0.22
	Incomplete^d^		

a,b = P < 0.001.

^a^The product experience is worse than expected.

^b^The product is better than expected.

^c^Actual liking moves towards the expectations.

^d^Assimilation occurs, but actual liking is lower than expectations.

Although no assimilation was observed in LTR lamb as no significant difference was detected between informed and perceived liking (blind acceptability) (P > 0.05), informed liking was higher for STR meat compared to perceived liking (P < 0.01): actual liking moved towards the expectations, thus demonstrating that the information on lamb geographical origin, transport duration, and welfare condition can affect the actual liking of the product. The effect of information can be explained on the basis of the assimilation model, as also shown in previous studies on consumers’ behaviour^41,42^, and consists in the shift of the informed liking of the product toward the direction of the expectations. In the present study, the information about geographical origin, transport duration, and welfare condition generated a positive impact on actual liking: the consumers assimilated their liking and they increased the actual acceptability towards the expectations. However, the assimilation was incomplete as a significant difference was detected between informed and actual liking (P < 0.05). This result is generally attributed to the role played by the sensory properties of the product in determining consumer acceptance. In a previous study, Napolitano *et al*.^9^ observed that a positive disconfirmation affected the informed liking, whereas a negative disconfirmation was unable to move the informed liking. In that study, the authors explained those different results based on the type of information which concerned the rearing system of lambs. In particular, they suggested that ewe-rearing was possibly considered the natural rearing conditions for lambs. In the present study, we observed that a negative disconfirmation affected the informed liking, whereas a positive disconfirmation was unable to affect the informed liking. Again, the consumers may perceive the importation of lambs, together with low welfare e long transport information, as the most common market situation and be rather affected by the positive information on the local origin of the product, higher welfare of the animals and consequently, short transport duration.

In the last 20 years, animal welfare has been recognized for consumers as the most important component of quality assurance of animal-based food^43^. A number of important attributes for animal-based food are also animal feeding, animal origin^2^, food appearance and price^44^. Napolitano *et al*.^9^ stated that if the meat is considered acceptable, in terms of sensory properties, the information about animal welfare can further increase meat acceptability, allowing the consumers to gain a more positive perception of meat. In the present study, the origin is confirmed to be an additional important extrinsic attribute that can influence the preferences of consumers, and their food purchase decision-making. The effect of the origin information is recognised to play a central role in affecting consumer decision of purchasing meat and meat products^18^, and it is affected by the aspects related to consumer’s beliefs, feelings or emotions, as suggested by Font-i-Furnols *et al*.^45^.

In the present study, the expectations due to the information on lamb geographical origin, transport duration, and welfare condition, positively influenced consumers’ product acceptability. In particular, the preference for meat derived from local lambs may be hypothesized to be associated with freshness, taste, quality and safety of the lamb meat, and feeling confident that consumers place in the local productive enterprises.

## Conclusion

Our results allow two main conclusions to be drawn. Firstly, long transport does not affect significant changes in meat quality indicators, such as pH, colour, mechanical properties and chemical composition. Accordingly, blood indicators reveale a response of adaptation to transport in terms of NEFA, CK, and white blood cells percentages, even if an increase of both haptoglobin and cortisol levels and a concomitant reduction of glucose level are observed. Secondly, consumers are affected by the information concerning short transport, local origin of lamb and good welfare moving their actual liking in the direction of expectancy. Therefore, the local production of lamb may sustain animal welfare and concomitantly sustain the domestic market, if the provenance is appropriately communicated to the consumers.

## Methods

### **Preliminary focus groups and food choice questionnaire**

Thirty participants were recruited after signing a consent form. The group consisted of 15 male and 15 female people with a mean age of 49.9 years. The education level ranged from secondary school (20%), to high school diploma (47%) and graduation (33%). Focus groups were conducted at the University of Basilicata in three different days with groups of 10 participants each by the same trained moderator, a 40-year old, female consumer scientist with a specific background on the focus and a decennial experience as focus group leader^46^. A semi-structured questionnaire was followed in order to be consistent across groups and, at the same time, allow for flexibility between groups^47^. The discussion conducted in each focus group was recorded and transcribed. Transcriptions were used to assess the perception of participants of aspects affecting their lamb choice.

The food choice questionnaire^48^ was administered to 101 consumers of lamb (51 female and 50 male people) with an education level ranging from secondary school (12%), to high school diploma (51%) and graduation (37%), and reporting to consume this product at least once a year. They were informed by a trained consumer about the aim of the study and the structure of the questionnaire, subsequently they filled the questionnaire autonomously but they could ask for clarifications to the interviewer.

The first section of the questionnaire consisted of items concerning the socio demographic characteristics of the consumers (gender, age, job category, education level), while the second section included items aimed at investigating the main aspects affecting their lamb choice. These items were identified during the preliminary focus groups. Each item was scored using a 7-point scale: from 1=unimportant to 7=very important.

### **Transport of lambs**

All animal procedures were approved by the Foggia University Institutional Animal Care and Use Committee (protocol number 003-2016) and were conducted under veterinary supervision. All applicable international, national, and/or institutional guidelines for the care and use of animals were followed (EU Directive 2010/63/EU). Thirty Merinos-derived male lambs were subjected to different transport distance and slaughtered at Foggia (Southern of Italy). Fifteen lambs were subjected to a short transport duration (STR), approximately around 1 h, starting from local farm to the slaughterhouse. A second group of fifteen lambs was subjected to a long transport (LTR) according to the Regulation EC 1/2005, around 22 h going through 1250 km, as long as the duration of transport from Bucharest (Romania) to the slaughterhouse located in Foggia. All of the animals were weaned and reared in the same conditions, including the feeding regimen based on a commercial concentrate having 16% crude protein and 11.4 ME/kg dry matter/day and free access to hay. The available space allowance was 0.32 m^2^ per lamb, in compliance with the EC Regulation 1/2005. During LTR the animals received water and straw ad libitum. At slaughterhouse, welfare issues (percentage of active animals, ambulatory animals, injuries, lameness, dead), as resulting from transport of lambs, were monitored by veterinarian.

Animals were slaughtered at 60 ± 5 days of age, according to industrial routines used in Italy and to the EU rule n. 1099/2009, after a lairage of approximately 12 hours with freely water availability and no access to feed. Each carcass was weighed and chilled at 1–3 °C. After 24 h *post-mortem* carcasses were split into two sides. The left side was used for meat quality measurements; *longissimus dorsi lumborum* (LDL) muscle was removed, sampled and then frozen at −20 °C. The right side was used for sensory analysis; LDL muscle was removed and was subsequently cut into steaks and then vacuum packed preserved at −20 °C until the day before panel evaluation.

### **Determination of metabolic and immune indicators in plasma**

Before slaughtering blood samples from each animal were collected from the jugular vein in vacuum tubes with and without Na heparin. Both plasma and serum samples were centrifuged at 2500 rpm for 10 min at room temperature and stored at −20 °C for subsequent analyses. Differential cell counts of neutrophils, lymphocytes, monocytes, eosinophils, and basophils, and haematocrit % were determined from blood samples using an automated cell counter (Cell-Dyn 3500 R, Abbott Diagnostics. CA). Packed-cell-volume (PCV) was obtained using the impedance method (ABX Pentra 60 C + Horiba). On serum samples, the measurement of glucose concentration (mg/dL), and creatine kinase (CK) activity (U/L) was carried out. On plasma samples, the measurement of concentrations of haptoglobin (mg/mL), cortisol (ng/mL), and NEFA (μmol/L) was performed.

Glucose concentrations were determined using the GOD-PAP test; whereas, plasma concentration of lactate was determined using LOD enzymatic test (HORIBA ABX S.A.S.). Creatinine kinase activity was measured by the UV-kinetic method optimized according to the Deutsche Gesellschaft für Klinische Chemie, in an autoanalyser (HORIBA ABX S.A.S.). Haptoglobin concentration was obtained by the peroxidise method, using a commercial kit Phase Haptoglobin (D.B.A. s.r.l, Italia). The intra- and interassay coefficients of variation were 0.945 mg/mL and 0.925 mg/mL, respectively. The sensitivity of test was 0.005 mg/mL. Cortisol concentrations were measured by immunoenzymatic test (kit Cortisol Assay R&D Systems, Italia). The intra- and interassay coefficients of variation were 4.10 ng/mL and 3.36 ng/mL, respectively. The sensitivity of test was 0.071 ng/mL.

### **Determination of Meat pH and Colour**

The pH was measured at 1 and 24 h post mortem using a portable pH meter (Crison Strumenti spa, Carpi, Italia) combined with glass electrode and inserted approximately two cm into LDL muscle.

Colour was measured using a colour meter Minolta CR 200 (D65: illuminant; Konica Minolta Sensing Inc., Sakai-ku, Sakai, Osaka, Japan) on 1 cm thick steaks from LDL. Before measurement, meat samples were allowed to bloom for 1 h at 3 ± 1 °C, stored in a plastic tray and over wrapped with a polyethylene film. The following CIE system colour coordinates (CIE, 1986) were measured: lightness (L*), redness (a*) and yellowness (b*) from three locations of the cut surface of the steaks. Chroma (C) and hue-angle (H) values were calculated as C = (a^2^ + b^2^)^0.5^ and H = tan^−1^(b/a), respectively. Final conversion of hue from radians to degrees was achieved by multiplying tan^−1^(b/a) by 180/π^49^.

### **Meat mechanical properties**

After thawing at 4 °C, Warner-Bratzler shear force (WBSF) and texture profile analysis (TPA) were tested on cooked meat. Steak samples (2.0 cm of thickness) were grill-cooked at 270 °C to rich a core temperature of 70 °C. Both instrumental measurements were conducted using an Instron 3343 universal testing machine with a 500 N load cell (Instron Ltd., High Wycombe, United Kingdom) as previously described by della Malva *et al*.^50^.

### **Meat chemical composition**

Each sample (50.0 g) was thawed and ground to homogeneous consistency using a food processor. Moisture, protein, lipid and ash contents in each sample were determined according to AOAC methods^51^.

### **Consumer test: perceived, expected and actual acceptability**

In order to setting up the consumer test, a number of 120 consumers were recruited in the city of Foggia (Apulia region, Southern Italy). All subjects were interviewed and were asked about their frequency of consumption of meat products at home (1 = never; 2 = once a year or less; 3 = 3–5 times a year; 4 = less than once a month; 5 = 1–2 times a month; 6 = more than 2 times a month; 7 = once a week). Eighty-two consumers were selected using predetermined screening criteria based on consumption of meat products with a frequency of at least once a month. In addition, consumers completed a form with personal data according to Napolitano *et al*.^9^. The main features of consumers are depicted in Table 7.

**Table 7:** Socio-demographic features of the subjects participating to the consumer test.

	Levels	Number	Percentage
Age	18–25 years	13	15.8
	26–35 years	28	34.2
	36–45 years	16	19.5
	46–55 years	25	30.5
Sex	Female	46	56.1
	Male	36	43.9
Education level	High School	20	24.4
	Graduated	15	18.3
	Post-graduated	47	57.3

The samples were thawed at 4 °C 24 h prior to evaluation. At the panel day, meat samples (3 ×3 ×2 cm, mean weight 50.3–55.6 g) were grilled (Maxima Grill electric MGRILL BIG) at 300 °C to an internal temperature of 75 °C assessed using a thermocouple probe inserted into the meat for about 12 min as described by Napolitano *et al*.^9^. Meat samples were offered to the subjects immediately after cooking.

The assessment of meat acceptability was planned in three tests according to Napolitano *et al*.^9^. In the first test, the consumers were offered meat samples from lambs subjected to short and long transport time in a random codified order of samples presentation directly in the plate. They were asked to taste the meat and rate their liking receiving no information on the products (Blind acceptability). In the second test, the subjects received a sheet with the information concerning the transport duration and its effect on animal welfare. They were asked to read carefully the information and give their liking expectation for that product (Expected acceptability). First and second tests were performed in the same day.

The third test was performed on the next day: the consumers were given meat from both STR and LTR group along with the information sheet. The consumers were instructed to read the information before tasting samples and express their liking score (Informed acceptability). Consumers rated their liking on a nine-point hedonic scale labelled at the left end with “extremely unpleasant”, at the right end with “extremely pleasant” and at the central point with “neither pleasant nor unpleasant”^52^.

In tests second (expectations produced by information) and third (acceptability generated by information and tasting of the product) the following information concerning the duration of transport and its effects on animal welfare were given to consumers:

1. STR: meat from local lambs subjected to a short transport time before slaughtering with a low impact on animal welfare.
2. LTR: meat from imported lambs subjected to a long transport time before slaughtering with an important impact on animal welfare.

In both days, for each session, eighty-two consumers were divided into groups and each animal from STR and LTR group was tested at least by 3 consumers. All meat from each animal and experimental group was tested.

### **Statistical analysis**

Nutritional, textural and quantitative descriptive sensory data were tested for normality using the Shapiro–Wilk test^53^; then, data were processed by ANOVA using the GLM procedure of SAS^54^. When significant effects were found (at P < 0.05), the Student t-test was used to locate significant differences between means.

The analysis of variance was carried out using the MIXED procedure of the SAS system for consumer panel test using the information condition: perceived (P), expected (E) and informed (I) liking, as fixed effect; whereas, consumers were included as random effect. To evaluate the effect of information on the consumer’s acceptability, the difference between perceived liking score and expected liking score (P-E) as well as differences between informed and perceived liking scores (I-P) and informed and expected liking scores (I-E) were calculated. Then, the Paired t-tests were performed in order to establish if those differences were significantly different from zero^55^.

## References

[1] Hersleth M, Næs T, Rødbotten M, Monteleone E (2012). Lamb meat-Importance of origin and grazing system for Italian and Norwegian consumers. Meat Sci..

[2] Bernués A, Olaizola A, Corcoran K (2003). Labelling information demanded by European consumers and relationships with purchasing motives, quality and safety of meat. Meat Sci..

[3] Bernabéu R, Rabadán A, El Orche NE, Díaz M (2018). Influence of quality labels on the formation of preferences of lamb meat consumers. A Spanish case study. Meat sci..

[4] Van der Lans IA, van Ittersum K, De Cicco A, Loseby M (2001). The role of the region of origin and EU certificates of origin in consumer evaluation of food products. Eur. Rev. Agric. Econ..

[5] Jordana J (2000). Traditional foods: Challenges facing the European food industry. Food Res. Int..

[6] Marino R (2017). “Consumers’ expectations and acceptability for low saturated fat “salami”: healthiness or taste?”. J. Sci. Food Agric..

[7] Guerrero L (2009). Consumer-driven definition of traditional food products and innovation in traditional foods. A qualitative cross-cultural study. Appetite.

[8] Sañudo C (2007). Regional variation in the hedonic evaluation of lamb meat from diverse production systems by consumers in six European countries. Meat Sci..

[9] Napolitano F (2007). Effect of information about animal welfare, expressed in terms of rearing conditions, on lamb acceptability. Meat Sci..

[10] Boutonnet JP (1999). Perspectives of the sheep meat world market on future production systems and trends. Small Rumin. Res..

[11] Broom DM (1986). Indicators of poor welfare. Br. Vet. J..

[12] Broom DM (1988). The scientific assessment of animal welfare. Appl. Anim. Behav. Sci..

[13] Miranda-de la Lama GC (2011). Effects of road type during transport on lamb welfare and meat quality in dry hot climates. Trop. Anim. Health Prod..

[14] Adenkola AY, Ayo JO (2010). Physiological and behavioural responses of livestock to road transportation stress: A review. Afr. J. Biotechnol..

[15] Dalmau A (2014). Effects of the duration of road transport on the physiology and meat quality of lambs. Anim. Prod. Sci..

[16] Swanson JC, Morrow-Tesch J (2001). Cattle transport: Historical, research, and future perspectives. J. Anim. Sci..

[17] Sanjuán-López AI, Philippidis G, Resano-Ezcaray H (2011). How useful is acceptability to explain economic value? An application on the introduction of innovative saffron products into commercial markets. Food Qual. Prefer..

[18] Braghieri A, Piazzolla N, Carlucci A, Bragaglio A, Napolitano F (2016). Sensory properties, consumer liking and choice determinants of Lucanian dry cured sausages. Meat Sci..

[19] Moskowitz HR (1995). Food quality: conceptual and sensory aspects. Food Qual. Prefer..

[20] Pieniak Z, Verbeke W, Vanhonacker F, Guerrero L, Hersleth M (2009). Association between traditional food consumption and motives for food choice in six European countries. Appetite.

[21] Napolitano F, Girolami A, Braghieri A (2010). Consumer liking and willingness to pay high welfare animal-based products. Trends Food Sci. Technol..

[22] Napolitano F, Pacelli C, Girolami A, Braghieri A (2008). Effect of information about animal welfare on consumer willingness to pay for yogurt. J. Dairy Sci..

[23] Kannan G (2000). Transportation of goats: Effects on physiological stress responses and live weight loss. J. Anim. Sci..

[24] Bórnez R, Linares MB, Vergara H (2009). Haematological, hormonal and biochemical blood parameters in lamb: Effect of age and blood sampling time. Livest. Sci..

[25] Ekiz B, Ergul Ekiz E, Kocak O, Yalcintan H, Yilmaz A (2012). Effect of pre-slaughter management regarding transportation and time in lairage on certain stress parameters, carcass and meat quality characteristics in Kivircik lambs. Meat Sci..

[26] Sapolsky RM, Romero M, Munck AU (2000). How do glucocorticoids influence stress responses? Integrating permissive, suppressive, stimulatory, and preparative actions. Endocr. Rev..

[27] Piñeiro M (2007). Characterisation of the pig acute phase protein response to road transport. Vet. J..

[28] Quaye IK (2008). Haptoglobin, inflammation and disease. Trans. R. Soc. Trop. Med. Hyg..

[29] Saeb M, Baghshani H, Nazifi S, Saeb S (2010). Physiological response of dromedary camels to road transportation in relation to circulating levels of cortisol, thyroid hormones and some serum biochemical parameters. Trop. Anim. Health Prod..

[30] Miranda-de la Lama GC (2010). Effect of the pre-slaughter logistic chain on some indicators of welfare in lambs. Livest. Sci..

[31] Broom DM, Gooder JA, Hall SJG, Lloyd DM, Parrot RF (1996). Hormonal and physiological effects of 15 hours journey in sheep: Comparison with the responses to loading, handling and penning in the absence of transport. Br. Vet. J..

[32] Knowles, T. & Warriss, P. Stress physiology of animals during transport. In Livestock Handling and Transport (ed. Grandin, T.) (Ed.), 312–328 (CABI, 2007).

[33] Zhong RZ, Liu HW, Zhou DW, Sun HX, Zhao CS (2011). The effects of road transportation on physiological responses and meat quality of sheep differing in age. J. Anim. Sci..

[34] De la Fuente J (2010). Physiological response and carcass and meat quality of suckling lambs in relation to transport time and stocking density during transport by road. Animal.

[35] Earley B (2012). The effect of transport by road and sea on physiology, immunity and behaviour of beef cattle. Res. Vet. Sci..

[36] Hall SJG, Broom DM, Kiddy GNS (1998). Effect of transportation on plasma cortisol and packed cell volume in different genotypes of sheep. Small Rumin. Res..

[37] Pritchard JC, Barr ARS, Whay HR (2010). Validity of a behavioural measure of heat stress and a skin tent test for dehydration in working horses and donkeys. Equine Vet. J..

[38] Lepherd ML, Canfield PJ, Hunt GB, Bosward KL (2009). Haematological, biochemical and selected acute phase protein reference intervals for weaned female Merino lambs. Austr. Vet. J..

[39] Marino R (2018). Effect of quinoa and/or linseed on immune response, productivity and quality of meat from merinos derived lambs. Animals.

[40] Devine CE (2006). Pre-slaughter stress arising from on-farm handling and its interactions with electrical stimulation on tenderness of lambs. Meat Sci..

[41] Cardello AV, Sawyer FM (1992). Effects of disconfirmed consumer expectations on food acceptance. J Sens Stud.

[42] Morales R, Aguiar APS, Subiabre I, Realini CE (2013). Beef acceptability and consumer expectations associated with production systemand marbling. Food Qual. Prefer..

[43] Montossi F (2013). Sustainable sheep production and consumer preference trends: Compatibilities, contradictions, and unresolved dilemmas. Meat Sci..

[44] Davidson A, Schröder MJA, Bower JA (2003). The importance of origin as a quality attribute for beef, results from a Scottish consumer survey. Int. J Consum. Stud..

[45] Font-i-Furnols M, Guerrero L (2014). Consumer preference, behaviour and perception about meat and meat products. An overview. Meat Sci..

[46] Krueger, R. A. Moderating focus groups. Thousand Oaks, CA: Sage. (1998).

[47] Krueger, R. A. Analyzing and reporting focus group results. Thousand Oaks, CA: Sage. (1998).

[48] Steptoe A, Pollard TM (1995). Development of a measure of the motives underlying the selection of food: the food choice questionnaire. Appetite.

[49] Liu Q, Scheller KK, Arp SC, Schaefer DM, Frigg M (1996). Color coordinates for assessment of dietary vitamin E effects on beef color stability. J Anim. Sci..

[50] della Malva A (2017). Proteomic approach to investigate the impact of different dietary supplementation on lamb meat tenderness. Meat Sci..

[51] AOAC methods. Official methods of analysis. Association of Official Analytical Chemists (1995).

[52] Kähkönen P, Tuorila H, Rita H (1996). How information enhances acceptability of a low-fat spread. Food Qual. Pref..

[53] Shapiro SS, Wilk MB (1965). An analysis of variance test for normality. Biometrika.

[54] SAS 2011. SAS/STAT User’s Guide (Version 9.2). Statistical Analysis System Inst, Cary, NC.

[55] Lange C, Rousseau F, Issanchou S (1999). Expectation, liking and purchase behaviour under economical constraint. Food Qual. Prefer..

